# PReS-FINAL-2272: Association of benign joint hypermobility syndrome with mitral valve prolapsed in Iranian children

**DOI:** 10.1186/1546-0096-11-S2-P262

**Published:** 2013-12-05

**Authors:** R Shiari, F Vaziri, E Zahmatkesh, H Javaherizadeh

**Affiliations:** 1 Pediatric Rheumatology, Shahid Beheshti University of Medical Sciences, Mofid Children's Hospital, Tehran, Iran, Islamic Republic Of; 2 Pediatrics, Shahid Beheshti University of Medical Sciences, Tehran, Iran, Islamic Republic Of; 3 Pediatrics, Ahvaz Jundishapur University, Tehran, Iran, Islamic Republic Of

## Introduction

Benign joint hypermobility syndrome (BJHS) is a clinical condition characterized by an increased ability of joints during passive and dynamic movements. Mitral valve prolapsed (MVP) is the most commonly diagnosed cardiac abnormality and affects around 5% of population. Abnormalities of collagen have been found in valves of patients with MVP.

## Objectives

There were limited published papers concerning children with BJHS and MVP. The aim of this study was to determine the association of BJHS with mitral valve prolapsed in children.

## Methods

Sixty-three children with benign joint hypermobility syndrome were included in case group and 63 without any rheumatologic disease were placed in control group. We used Carter-Wilkinson and Beighton criteria for diagnosing of benign joint hypermobility syndrome. MVP was evaluated by echocardiography in both groups. The mitral leaflet displacement >2 mm considered as cut off for diagnosis of MVP.

## Results

In this study, 32 girls and 31 boys were included. Mean of age in case group was 7.1 was 6.9 (p = 0.001). Mitral valve prolapse was significantly higher among cases with BJHS aged >7 (58.8%) year compared to 3-7 (41.2%) year of age (p = 0.027). Heart murmur and palpitation was more common among children with benign joint hypermobility syndrome with MVP compared to children without MVP (p < 0.05).

## Conclusion

The incidence of MVP among children with benign joint hypermobility was significantly higher than control group.

## Disclosure of interest

None declared.

